# Rare Lumbar Epidural Lesions: Differentiating Chronic Spontaneous Lumbar Epidural Hematoma from Lumbar Synovial Cyst—A Single-Center Comparative Study

**DOI:** 10.3390/medicina62091703

**Published:** 2026-09-05

**Authors:** Nimetullah Alper Durmuş, Haytham Jabarin, Ali Şahin, Eray Abat, Ahmet Küçük, Şükrü Oral

**Affiliations:** 1 Department of Neurosurgery, Faculty of Medicine, Erciyes University, Kayseri 38030, Turkey; nimetullahalper@hotmail.com (N.A.D.); dralishn@gmail.com (A.Ş.); ahmetkucuk@erciyes.edu.tr (A.K.); 2 Department of Neurosurgery, Kayseri State Hospital, Kayseri 38010, Turkey; jabarin.h@gmail.com; 3 Department of Neurosurgery, Develi Dr. Ekrem Karakaya State Hospital, Kayseri 38400, Turkey; eray_abat@hotmail.com

**Keywords:** chronic spontaneous lumbar epidural hematoma, lumbar synovial cyst, differential diagnosis, magnetic resonance imaging, lumbar decompression, surgical outcomes

## Abstract

*Background and Objectives:* Chronic spontaneous lumbar epidural hematoma (CSLEH) is a rare condition that may mimic a lumbar synovial cyst clinically and radiologically, making accurate preoperative diagnosis challenging. This study aimed to compare the clinical and magnetic resonance imaging (MRI) characteristics of CSLEHs and lumbar synovial cysts and to identify features that may facilitate their preoperative differentiation. *Materials and Methods:* This retrospective single-center study included 16 adult patients who underwent surgical decompression for symptomatic extradural lesions located between T12 and S1 and were ultimately diagnosed with either CSLEH or lumbar synovial cyst. Based on definitive intraoperative findings with histopathological confirmation when applicable, patients were classified into a CSLEH group (*n* = 6) or a lumbar synovial cyst group (*n* = 10). Demographic characteristics, clinical presentation, MRI findings, lesion localization, neurological deficits, pain severity assessed using the Visual Analog Scale (VAS), and postoperative outcomes were compared between the groups. *Results:* Patients with CSLEHs had a significantly shorter symptom duration (*p* = 0.001), more frequent anticoagulant use (*p* = 0.008), and a higher prevalence of motor weakness (*p* < 0.001) and sensory deficits (*p* = 0.001) than those with lumbar synovial cysts. Preoperative pain severity was significantly greater in the CSLEH group (*p* < 0.001). No significant differences were observed in MRI signal characteristics or lesion distribution between the groups. Surgical decompression was associated with marked postoperative pain improvement in both groups, with a greater reduction in VAS scores (ΔVAS) in the CSLEH group (*p* = 0.007). No postoperative complications were observed. *Conclusions:* CSLEH should be considered in the differential diagnosis of lumbar synovial cyst, particularly in patients with a relatively short symptom duration, anticoagulant use, and progressive neurological deficits. Although conventional MRI remains essential for preoperative evaluation, substantial overlap in MRI characteristics may limit reliable differentiation between these entities. Integration of clinical and radiological findings may improve preoperative diagnostic suspicion, while surgical exploration can provide both definitive diagnosis and effective neural decompression.

## 1. Introduction

Lumbar synovial cysts are uncommon degenerative juxtafacet lesions closely associated with degeneration of the lumbar facet joints and are increasingly recognized as a cause of lumbar radiculopathy and spinal canal stenosis in the elderly population [1,2,3].

Lumbar synovial cysts are rare, with a reported incidence of less than 0.5% in the symptomatic population [2]. These lesions are being recognized with increasing frequency owing to the widespread use of magnetic resonance imaging (MRI), although they remain relatively uncommon [2,3]. They are generally considered part of the spectrum of degenerative lumbar spine disease and are closely associated with facet joint arthropathy, segmental instability, and degenerative spondylolisthesis [2,4,5]. Although these lesions may occur at any lumbar level, the L4–L5 segment is most frequently affected because of its greater mobility and susceptibility to degenerative changes [2,3]. Clinical manifestations vary according to the degree of neural compression and commonly include low back pain, unilateral or bilateral radiculopathy, neurogenic claudication, and, less frequently, progressive neurological deficits or cauda equina syndrome [4,6].

Magnetic resonance imaging (MRI) is considered the imaging modality of choice because of its excellent soft-tissue contrast and its ability to define the relationship between the lesion, facet joint, neural structures, and surrounding soft tissues [2,3]. Typical synovial cysts appear as well-defined extradural lesions adjacent to the degenerated facet joint, with variable signal characteristics depending on their proteinaceous content, calcification, or hemorrhagic transformation [7,8]. Consequently, atypical MRI appearances may lead to an incorrect preoperative diagnosis, making the differential diagnosis between lumbar synovial cysts and other extradural lesions particularly challenging [9,10,11]. The differential diagnosis of lumbar extradural lesions includes sequestered or migrated disc fragments, epidural hematomas, epidural abscesses, neoplasms, and synovial or ganglion cysts, which may demonstrate overlapping clinical and MRI characteristics [9,10,11].

Spontaneous spinal epidural hematoma (SSEH) is an uncommon spinal emergency, whereas its chronic lumbar form represents an exceptionally rare entity [12,13,14]. SSEH accounts for less than 1% of spinal canal space-occupying lesions, with an estimated annual incidence of approximately 0.1 cases per 100,000 persons [14]. Chronic lumbar SSEH is considerably rarer; a previous literature review identified only 20 reported cases [12]. Chronic spontaneous lumbar epidural hematoma (CSLEH) is characterized by a more indolent clinical course and gradually progressive symptoms, which may closely resemble those of degenerative lumbar disorders [10,12,15]. Although the exact etiology of SSEH remains uncertain in many cases, several predisposing factors have been reported, including anticoagulant therapy, coagulation disorders, vascular malformations, hypertension, and conditions associated with increased venous pressure [16]. Both venous and arterial sources of epidural bleeding have been proposed, although the underlying mechanism remains incompletely understood [16]. Repeated episodes of minor bleeding, subsequent hematoma organization, and progressive degradation of blood products may produce heterogeneous MRI signal characteristics that substantially overlap with those of lumbar synovial cysts and other extradural lesions, thereby complicating accurate preoperative diagnosis [7,8,10].

Previous reports have highlighted the diagnostic difficulty associated with chronic spinal epidural hematomas. Kanematsu et al. described the radiological features and clinical course of four patients with chronic spinal epidural hematoma [10], while Okazaki et al. reported an idiopathic chronic lumbar epidural hematoma and reviewed previously reported cases [12]. Matsui et al. also described a chronic spontaneous lumbar epidural hematoma that radiologically mimicked an extradural spinal tumor [15]. These reports illustrate the variable radiological appearance of chronic epidural hematomas and their potential to mimic other extradural spinal lesions.

Although lumbar synovial cysts and chronic spontaneous lumbar epidural hematomas have each been described in case reports, case series, and review articles, the available literature has largely addressed these entities separately [10,12,16]. Despite increasing recognition of both conditions, direct comparative studies evaluating their clinical presentation, MRI findings, and surgical outcomes are lacking [10,17].

Therefore, the aim of this study was to compare the clinical and MRI characteristics of chronic spontaneous lumbar epidural hematomas and lumbar synovial cysts and to identify features that may facilitate their preoperative differentiation.

## 2. Materials and Methods

### 2.1. Study Design and Patient Selection

This retrospective observational study was conducted at the Department of Neurosurgery of a single tertiary referral university hospital. The study was designed to evaluate lumbar extradural lesions initially suspected to represent lumbar synovial cysts and to compare the clinical, radiological, and postoperative characteristics of patients ultimately diagnosed with chronic spontaneous lumbar epidural hematoma or lumbar synovial cyst. The study was reported in accordance with the Strengthening the Reporting of Observational Studies in Epidemiology (STROBE) guidelines [18]. Ethical approval was obtained from the Erciyes University Ethics Committee (approval no. 2025/474; 8 October 2025), and the study was conducted in accordance with the Declaration of Helsinki and its subsequent amendments.

Medical records of patients who underwent lumbar decompression surgery between February 2021 and September 2025 were retrospectively reviewed. Patients with symptomatic extradural lesions located between T12 and S1 who underwent surgical decompression and were ultimately diagnosed with either chronic spontaneous lumbar epidural hematoma or a lumbar synovial cyst were screened for eligibility. After application of the predefined inclusion and exclusion criteria, all eligible patients were included in the analysis. The final cohort comprised 16 patients: 6 with chronic spontaneous lumbar epidural hematoma and 10 with lumbar synovial cyst. Inclusion criteria comprised age ≥ 18 years, availability of complete demographic, clinical, radiological, operative, and postoperative follow-up data, and confirmation of either chronic spontaneous lumbar epidural hematoma or a lumbar synovial cyst based on intraoperative findings, with histopathological confirmation when applicable. Patients with incomplete records, traumatic spinal epidural hematoma, spinal infections, tumors, previous surgery at the same lumbar level were excluded from the analysis.

### 2.2. Clinical and Radiological Evaluation

Demographic and baseline clinical variables collected from institutional electronic medical records included age, sex, presence of comorbid diseases, anticoagulant use, duration of symptoms before hospital admission, motor deficits, and sensory deficits. Symptom duration was defined as the interval between onset of symptoms and hospital presentation requiring neurosurgical evaluation. Motor deficit was recorded when objective weakness was documented during neurological examination, whereas sensory deficit was defined as clinically documented sensory disturbance corresponding to the affected neural distribution.

Radiological assessment was performed using preoperative lumbar magnetic resonance imaging (MRI) studies. Preoperative MRI examinations were reviewed by the treating neurosurgical team as part of routine clinical evaluation.

MRI characteristics evaluated included signal intensity patterns on T1-weighted and T2-weighted sequences, lesion localization within the lumbar spine, and the anatomical relationship of the lesion to the adjacent facet joint. Signal characteristics were categorized as hypointense, isointense, or hyperintense on routine T1- and T2-weighted MRI sequences. Lesion localization was recorded according to the involved spinal level (T12–L1, L1–L2, L2–L3, L3–L4, L4–L5, and L5–S1). The relationship with the facet joint was assessed based on direct anatomical continuity or adjacency of the lesion to the corresponding facet joint on preoperative MRI. Facet joint association was considered a characteristic imaging feature of lumbar synovial cysts, consistent with their typical juxtafacet origin [19].

### 2.3. Surgical and Outcome Assessment

All patients underwent lumbar decompression surgery according to institutional neurosurgical practice standards with the objective of relieving neural compression caused by symptomatic extradural lesions. Final diagnosis was established intraoperatively. Histopathological examination confirmed the diagnosis in patients with lumbar synovial cysts when tissue specimens were available, whereas the diagnosis of chronic spontaneous lumbar epidural hematoma was established based on characteristic intraoperative findings. Postoperative outcomes included preoperative and postoperative pain severity, magnitude of pain improvement, neurological recovery, and occurrence of postoperative complications. Neurological recovery was assessed from postoperative clinical records based on documented improvement in preoperative motor deficits; however, the timing of postoperative neurological assessment was not standardized across patients.

Pain intensity was assessed using the Visual Analog Scale (VAS), ranging from 0 (no pain) to 10 (worst imaginable pain), with higher scores indicating greater pain intensity [20]. Pain improvement was calculated as the difference between preoperative and postoperative VAS scores (ΔVAS = preoperative VAS − postoperative VAS).

### 2.4. Statistical Analysis

Statistical analyses were performed using IBM SPSS Statistics for Windows, Version 22.0 (IBM Corp., Armonk, NY, USA). Continuous variables were initially assessed for normality using the Shapiro–Wilk test due to the limited sample size. Variables demonstrating approximately normal distribution were reported as mean ± standard deviation (SD), whereas non-parametric analyses were applied to variables not meeting normality assumptions. Continuous variables were compared using the independent-samples *t* test or the Mann–Whitney U test, as appropriate. Categorical variables were expressed as frequencies and percentages and compared using Fisher’s exact test or the exact chi-square test, as appropriate. To complement *p* values and provide information on the magnitude and precision of between-group differences, effect sizes with 95% confidence intervals (CIs) were additionally calculated. Hedges’ g was used for continuous variables, whereas absolute differences in proportions were used for categorical variables. A two-sided *p* value < 0.05 was considered statistically significant. Given the limited number of patients and the exploratory nature of the analysis, effect estimates and their 95% CIs were interpreted cautiously, and multivariable regression modeling was not performed to minimize instability and potential overfitting. No a priori sample size calculation was performed because of the retrospective design and the rarity of CSLEH; instead, all eligible patients treated during the predefined study period were included.

## 3. Results

### 3.1. Clinical Characteristics

A total of 16 patients who underwent surgical decompression for symptomatic extradural lesions located between T12 and S1 were included in the study. Based on definitive intraoperative and/or histopathological findings, 6 patients were diagnosed with chronic spontaneous lumbar epidural hematomas (CSLEH, *n* = 6) and 10 with lumbar synovial cysts (*n* = 10).

Baseline demographic characteristics were comparable between the two groups (Table 1). Although patients with CSLEH tended to be older than those with lumbar synovial cysts (66.8 ± 7.3 vs. 59.8 ± 11.9 years), the difference was not statistically significant (*p* = 0.216). Similarly, no significant differences were observed in sex distribution or the prevalence of comorbidities between the groups (*p* = 0.607 and *p* = 0.588, respectively).

Several clinical characteristics differed significantly between the two groups (Table 1). Anticoagulant use was more frequent in patients with CSLEH than in those with lumbar synovial cysts (83.3% vs. 10.0%, *p* = 0.008). Symptom duration was significantly shorter in the CSLEH group (23.8 ± 12.3 vs. 128.5 ± 73.7 days, *p* = 0.001). Motor weakness was present in all patients with CSLEH but in none of the patients with lumbar synovial cysts (100.0% vs. 0.0%, *p* < 0.001). Sensory deficits were also more common in the CSLEH group, occurring in 83.3% of patients, whereas none of the patients with lumbar synovial cysts had sensory deficits (*p* = 0.001). Baseline demographic and clinical comparisons between groups are summarized in Table 1.

### 3.2. Radiological Findings

Radiological findings are summarized in Table 2. T1-weighted MRI demonstrated predominantly isointense lesions in both groups. All CSLEH lesions were isointense on T1-weighted imaging, whereas hypointense lesions were identified in 30.0% of lumbar synovial cysts; however, this difference was not statistically significant (*p* = 0.250). On T2-weighted imaging, hyperintense signal intensity predominated in both groups, although iso- and hypointense signals were observed only in the CSLEH group. The distribution of T2-weighted signal characteristics did not differ significantly between the groups (*p* = 0.125). All lumbar synovial cysts demonstrated an anatomical relationship with the adjacent facet joint on preoperative MRI, consistent with their typical juxtafacet location.

Analysis of lesion localization demonstrated that CSLEH lesions were distributed across several lumbar levels, including L1–L2, L2–L3, and L3–L4, whereas lumbar synovial cysts were predominantly located at L4–L5 (60.0%). Lesions at L5–S1 were observed exclusively in the synovial cyst group. Overall, lesion localization did not differ significantly between the groups (*p* = 0.126).

**Figure 1:**
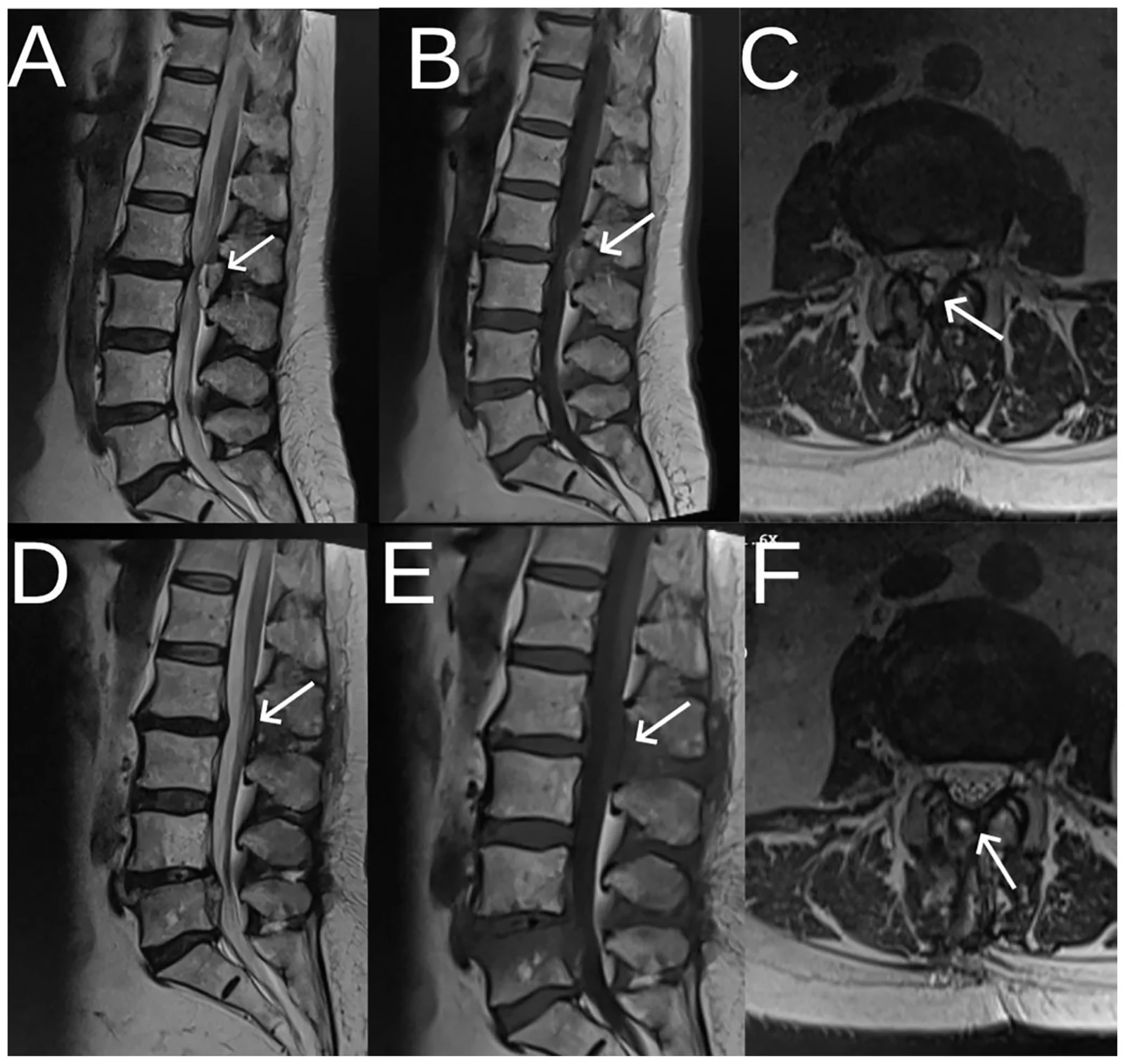
Representative case of chronic spontaneous lumbar epidural hematoma at the L2–L3 level. (**A**) Preoperative sagittal T2-weighted MRI demonstrating a dorsal epidural lesion at the L2–L3 level causing marked compression of the thecal sac (arrow). (**B**) Preoperative sagittal T1-weighted MRI demonstrating the dorsal epidural lesion (arrow). (**C**) Preoperative axial T2-weighted MRI demonstrating significant spinal canal compression by the epidural lesion (arrow). (**D**) Postoperative sagittal T2-weighted MRI demonstrating complete evacuation of the hematoma and satisfactory decompression of the thecal sac (arrow). (**E**) Postoperative sagittal T1-weighted MRI demonstrating no residual epidural lesion following surgical evacuation (arrow). (**F**) Postoperative axial T2-weighted MRI demonstrating adequate spinal canal decompression following hematoma evacuation (arrow).

**Figure 2:**
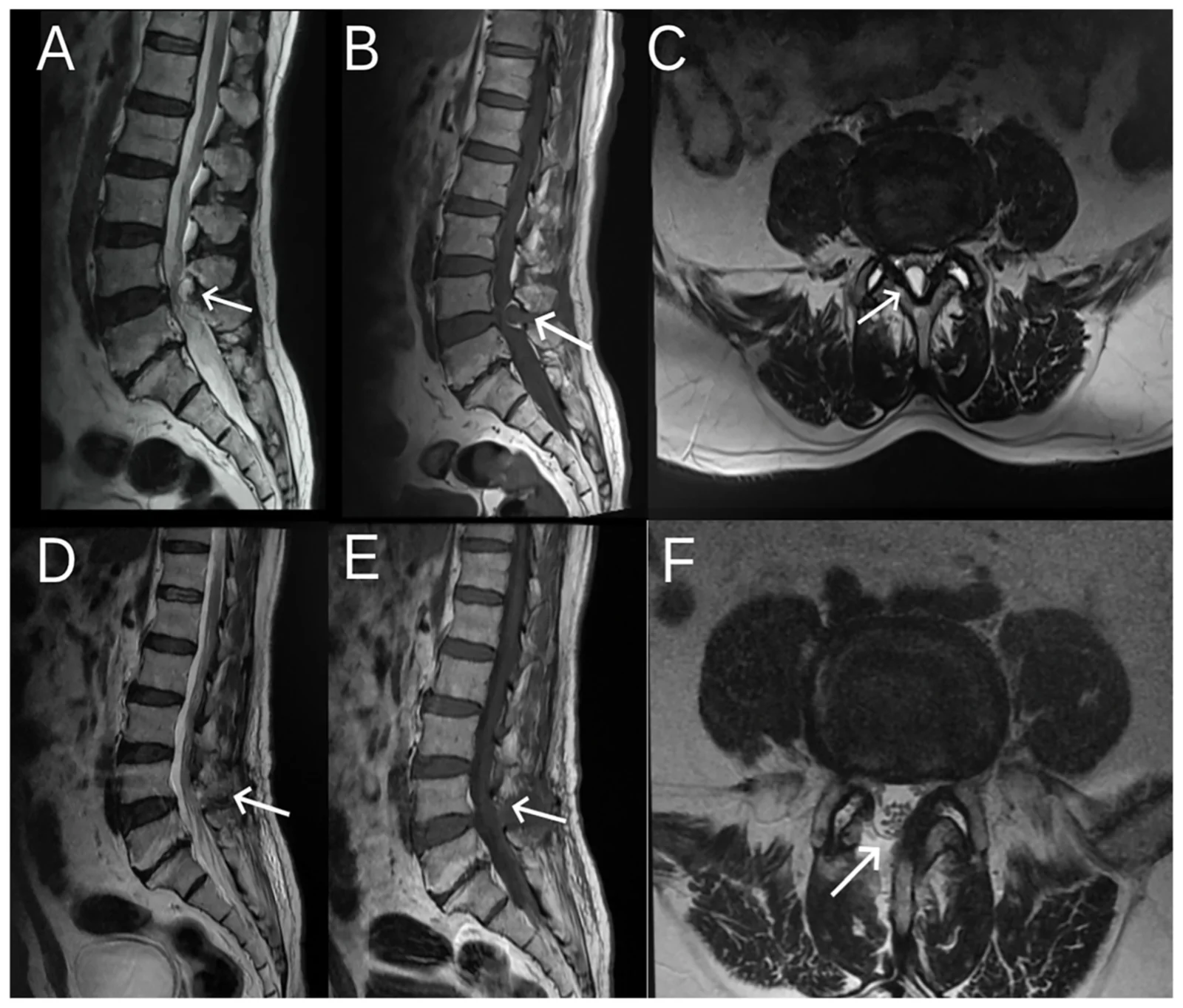
Representative case of chronic spontaneous lumbar epidural hematoma at the L4–L5 level. (**A**) Preoperative sagittal T2-weighted MRI demonstrating a dorsal epidural lesion at the L4–L5 level causing marked compression of the thecal sac (arrow). (**B**) Preoperative sagittal T1-weighted MRI demonstrating the dorsal epidural lesion (arrow). (**C**) Preoperative axial T2-weighted MRI demonstrating significant spinal canal compression by the epidural lesion (arrow). (**D**) Postoperative sagittal T2-weighted MRI demonstrating complete evacuation of the hematoma and satisfactory decompression of the thecal sac (arrow). (**E**) Postoperative sagittal T1-weighted MRI demonstrating no residual epidural lesion following hematoma evacuation (arrow). (**F**) Postoperative axial T2-weighted MRI demonstrating adequate spinal canal decompression following hematoma evacuation (arrow).

**Figure 3:**
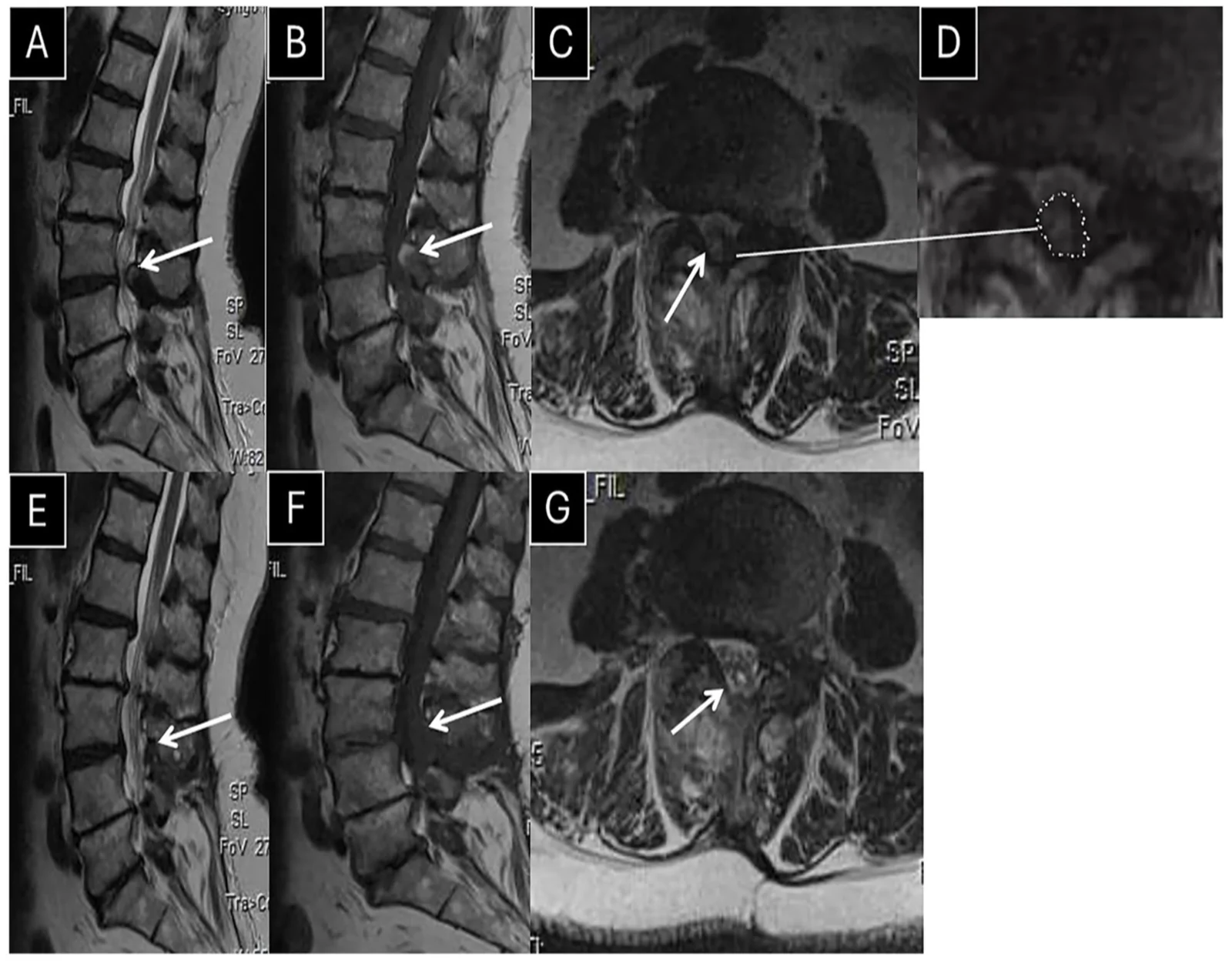
Representative case of chronic spontaneous lumbar epidural hematoma at the L3–L4 level. (**A**) Preoperative sagittal T2-weighted MRI demonstrating a dorsal epidural lesion at the L3–L4 level causing marked compression of the thecal sac (arrow). (**B**) Preoperative sagittal T1-weighted MRI demonstrating the dorsal epidural lesion (arrow). (**C**) Preoperative axial T2-weighted MRI demonstrating significant spinal canal compression by the epidural lesion (arrow). (**D**) Magnified view of panel C demonstrating the dorsal epidural lesion (dashed circle). (**E**) Postoperative sagittal T2-weighted MRI demonstrating complete evacuation of the hematoma and satisfactory decompression of the thecal sac (arrow). (**F**) Postoperative sagittal T1-weighted MRI demonstrating no residual epidural lesion following hematoma evacuation (arrow). (**G**) Postoperative axial T2-weighted MRI demonstrating adequate spinal canal decompression following hematoma evacuation (arrow).

**Figure 4:**
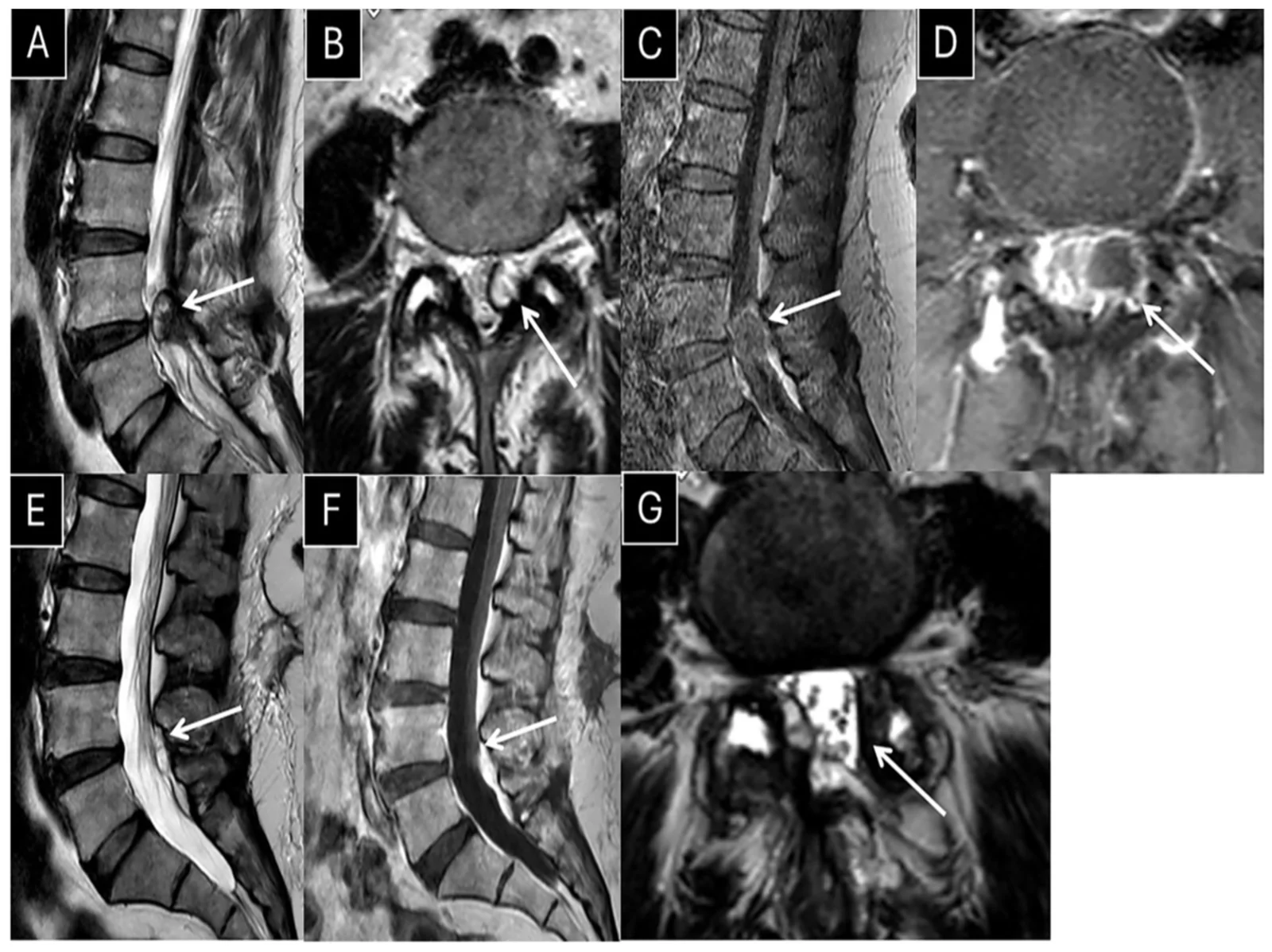
Representative case of lumbar synovial cyst at the L4–L5 level. (**A**) Preoperative sagittal T2-weighted MRI demonstrating a lumbar synovial cyst causing marked compression of the thecal sac (arrow). (**B**) Preoperative axial T2-weighted MRI demonstrating spinal canal compression by the lumbar synovial cyst (arrow). (**C**) Preoperative sagittal contrast-enhanced T1-weighted MRI demonstrating the lumbar synovial cyst (arrow). (**D**) Preoperative axial contrast-enhanced T1-weighted MRI demonstrating the lumbar synovial cyst adjacent to the facet joint (arrow). (**E**) Postoperative sagittal T2-weighted MRI demonstrating complete excision of the cyst and satisfactory decompression of the thecal sac (arrow). (**F**) Postoperative sagittal contrast-enhanced T1-weighted MRI demonstrating no residual lumbar synovial cyst (arrow). (**G**) Postoperative axial T2-weighted MRI demonstrating adequate spinal canal decompression following cyst excision (arrow).

Representative preoperative and postoperative MRI findings of patients with chronic spontaneous lumbar epidural hematomas and lumbar synovial cysts are presented in Figure 1, Figure 2, Figure 3 and Figure 4.

Analysis of lesion localization demonstrated that CSLEH lesions were distributed across several lumbar levels (L1–L2 and L3–L4), whereas lumbar synovial cysts were predominantly located at L4–L5 (60.0%). Lesions at L5–S1 were observed exclusively in the synovial cyst group. Overall, lesion localization did not differ significantly between the groups (*p* = 0.126) (Table 2).

### 3.3. Surgical and Postoperative Outcomes

**Figure 5:**
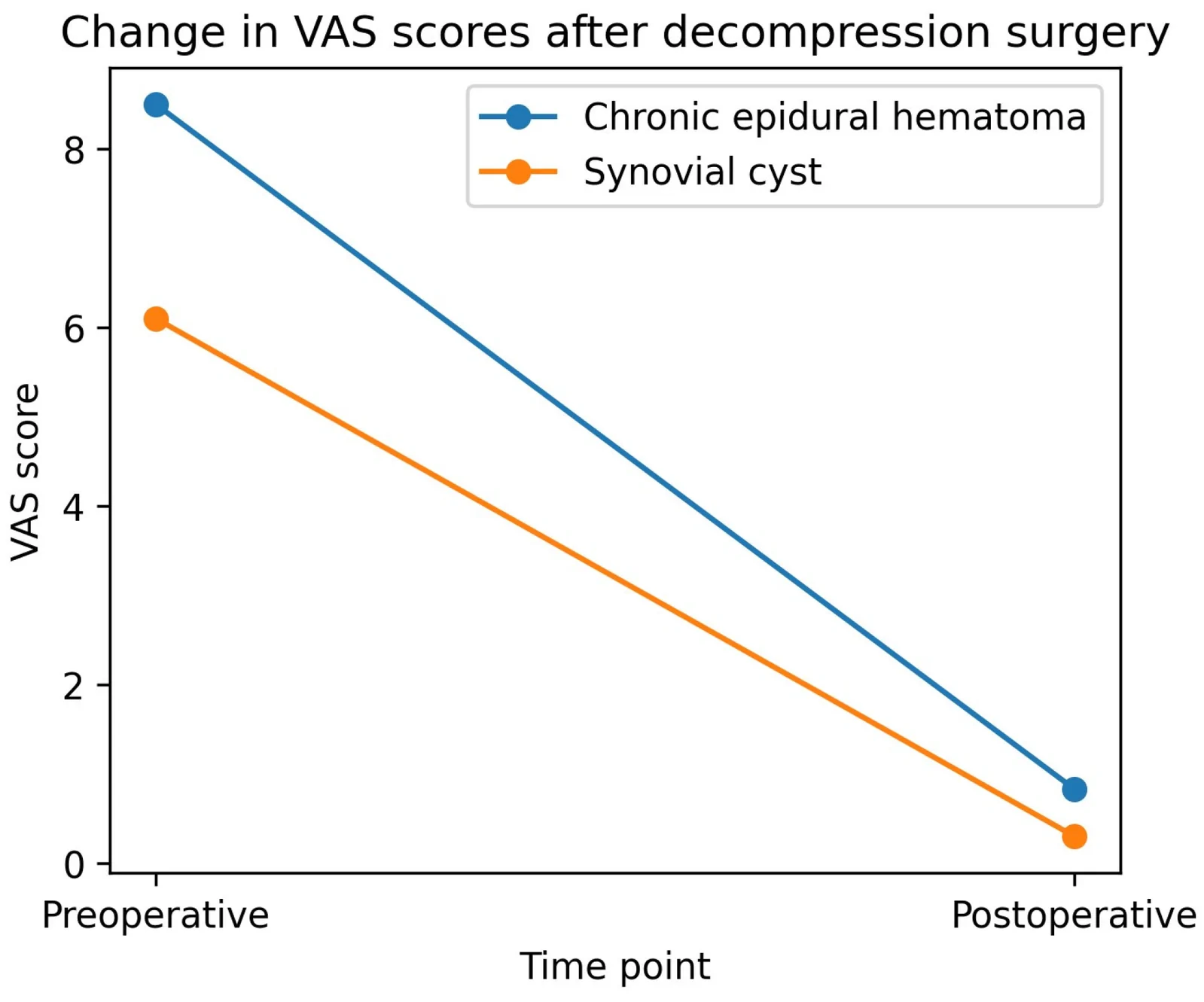
Comparison of preoperative and postoperative visual analog scale (VAS) pain scores in patients with chronic spontaneous lumbar epidural hematoma and lumbar synovial cyst.

Postoperative outcomes are summarized in Table 3, and changes in pain severity before and after surgery are illustrated in Figure 5. Patients with CSLEH had significantly greater preoperative pain severity than those with lumbar synovial cysts (VAS 8.5 ± 0.5 vs. 6.1 ± 1.0, *p* < 0.001). Following surgical decompression, postoperative VAS scores decreased markedly in both groups, with no significant difference between the groups (0.8 ± 0.8 vs. 0.3 ± 0.5, *p* = 0.181). The magnitude of pain improvement (ΔVAS) was significantly greater in the CSLEH group than in the lumbar synovial cyst group (7.7 ± 1.2 vs. 5.8 ± 0.8, *p* = 0.007). All six patients with CSLEH who presented with preoperative motor weakness achieved complete motor recovery during postoperative follow-up. No postoperative complications were observed in either group.

Comparisons of surgical outcomes and postoperative pain changes are shown in Table 3, while temporal changes in pain scores following decompression surgery are illustrated in Figure 5. Postoperative complications were defined as any adverse clinical event documented during postoperative follow-up.

## 4. Discussion

The present study demonstrates that chronic spontaneous lumbar epidural hematomas (CSLEH) can closely mimic lumbar synovial cysts both clinically and radiologically, posing a considerable challenge for accurate preoperative diagnosis. Compared with patients with lumbar synovial cysts, those with CSLEH exhibited significantly shorter symptom duration, more frequent anticoagulant use, a higher prevalence of motor weakness and sensory deficits, and greater preoperative pain severity. Despite these marked clinical differences, conventional MRI findings showed substantial overlap between the two entities, limiting reliable preoperative differentiation. Nevertheless, surgical decompression resulted in favorable postoperative outcomes in both groups while also establishing the definitive diagnosis. Collectively, these findings emphasize the importance of integrating clinical presentation with radiological assessment to improve the preoperative recognition of CSLEH in patients with suspected lumbar synovial cysts.

Preoperative differentiation between lumbar synovial cysts and chronic spontaneous lumbar epidural hematomas remains challenging because these entities often exhibit considerable overlap in both their clinical presentation and MRI characteristics. Lumbar synovial cysts are degenerative juxtafacet lesions that develop in association with facet joint arthropathy, segmental instability, and degenerative spondylolisthesis, whereas chronic spontaneous lumbar epidural hematomas are thought to develop through repeated episodes of minor bleeding followed by progressive hematoma organization and fibrosis [5,7,8,10]. Despite their distinct pathophysiological mechanisms, both entities may produce extradural space-occupying lesions that compress adjacent neural structures and manifest with similar symptoms, including low back pain, radiculopathy, neurogenic claudication, and, in advanced cases, neurological deficits [4,6,10]. Consequently, clinical presentation alone is frequently insufficient for reliable preoperative differentiation, particularly when MRI signal characteristics are modified by hemorrhagic transformation, blood product evolution, or chronic hematoma organization [7,8,10].

In the present study, conventional MRI findings did not reliably differentiate chronic spontaneous lumbar epidural hematoma from lumbar synovial cyst. Although isointense T1-weighted signals predominated in both groups and hyperintense T2-weighted signals represented the most common imaging pattern, no statistically significant differences were identified in MRI signal characteristics between the two entities. Similarly, lesion localization did not differ significantly between the groups. These findings suggest that conventional MRI alone may be insufficient for accurate preoperative differentiation.

An additional radiological feature considered during preoperative evaluation was the relationship of the lesion to the facet joint. In our series, all lumbar synovial cysts were associated with the adjacent facet joint, consistent with their typical juxtafacet origin. This anatomical relationship may therefore serve as a useful supportive imaging feature favoring a lumbar synovial cyst when evaluating an otherwise nonspecific lumbar extradural lesion. However, the relationship to the facet joint should be interpreted in conjunction with the clinical presentation and other MRI characteristics rather than as an isolated diagnostic criterion.

Previous studies have likewise demonstrated that the MRI appearance of both lumbar synovial cysts and chronic epidural hematomas is highly variable and is influenced by hemorrhagic transformation, blood product degradation, and the stage of hematoma organization, all of which may substantially alter MRI signal characteristics [7,8,10]. Hemorrhagic degeneration within synovial cysts may produce T1 hyperintensity, heterogeneous T2 signal intensity, peripheral hemosiderin deposition, or contrast enhancement, whereas chronic epidural hematomas may exhibit comparable MRI appearances during different stages of hemoglobin degradation, resulting in considerable radiological overlap [7,8,10]. This radiological overlap is further compounded by the fact that both lesions most commonly occur in the lower lumbar spine, particularly at the L4–L5 level, although chronic epidural hematomas may occasionally occur at more cranial levels [7,8,10]. Taken together, MRI findings should be interpreted in conjunction with the patient’s clinical presentation, neurological examination, and relevant medical history rather than in isolation, particularly in patients in whom chronic spontaneous lumbar epidural hematoma is considered a potential differential diagnosis of a lumbar synovial cyst [10,12,15,16].

In the present study, patients with chronic spontaneous lumbar epidural hematoma presented with a significantly shorter symptom duration, more frequent anticoagulant use, and a markedly higher prevalence of motor and sensory deficits than patients with lumbar synovial cyst.

These findings suggest that, despite their similar radiological appearance, CSLEH tends to present with more severe neurological impairment than a lumbar synovial cyst and prompt careful evaluation of the differential diagnosis.

Previous reports have consistently shown that spontaneous spinal epidural hematomas typically present with acute or subacute onset of severe pain followed by rapidly progressive neurological deterioration, frequently requiring urgent surgical decompression [10,12,14,16]. In contrast, lumbar synovial cysts usually develop gradually as a consequence of chronic degenerative changes and most commonly manifest with slowly progressive radicular pain, neurogenic claudication, or chronic low back pain, whereas objective neurological deficits are relatively uncommon [2,3,4,6]. Although the chronic form of spontaneous lumbar epidural hematoma may exhibit a more indolent clinical course than classic SSEH, progressive neurological impairment has been consistently reported, particularly in patients with recurrent minor hemorrhage and progressive hematoma organization [8,10,12,15].

The markedly higher frequency of anticoagulant use observed in our CSLEH cohort is consistent with the proposed role of repeated minor bleeding in the pathogenesis of chronic epidural hematoma. Previous case reports and small case series have suggested that anticoagulant therapy may increase the risk of spontaneous spinal epidural bleeding by facilitating repeated minor hemorrhagic events, thereby contributing to chronic hematoma formation, although the exact mechanism remains incompletely understood [10,12,13,14,15]. Accordingly, a history of anticoagulant use, particularly when accompanied by rapidly progressive neurological deficits, should raise clinical suspicion for CSLEH in patients with a presumed diagnosis of a lumbar synovial cyst (Table 4).

Despite presenting with more severe preoperative neurological impairment and higher pain severity, patients with CSLEH experienced favorable postoperative outcomes following surgical decompression. In the present study, postoperative pain scores improved significantly in both groups, and no significant differences were observed in postoperative VAS scores or complication rates. Furthermore, the magnitude of pain improvement was significantly greater in the CSLEH group, indicating that surgical decompression may achieve substantial symptomatic recovery despite a more severe clinical presentation.

These findings are consistent with previous reports indicating that surgical decompression remains the treatment of choice for both symptomatic lumbar synovial cysts and spinal epidural hematomas [2,3,4,6,10,12,13,15]. In patients with spontaneous spinal epidural hematoma, neurological recovery is closely associated with early diagnosis and prompt decompression, whereas delayed intervention has been linked to poorer functional outcomes [12,13,14,15]. Similarly, surgical excision of symptomatic lumbar synovial cysts has been reported to provide durable pain relief and neurological improvement with low recurrence and complication rates [2,3,4,6].

Although our cohort was relatively small, the absence of postoperative complications and the marked improvement in pain observed in both groups further support the effectiveness of surgical decompression in appropriately selected patients. These findings also emphasize that, despite the diagnostic uncertainty frequently encountered before surgery, surgical management establishes the definitive diagnosis while providing effective treatment.

The principal strength and novelty of the present study is that, to our knowledge, it represents the first direct comparative analysis of chronic spontaneous lumbar epidural hematoma and lumbar synovial cyst. Previous reports have predominantly described these entities separately as case reports, small case series, or review articles, without directly comparing their clinical and radiological characteristics [10,12,15,16]. In contrast, the present study directly compares demographic characteristics, clinical presentation, MRI findings, intraoperative diagnosis, and postoperative outcomes between the two entities within a single cohort. This comparative approach identifies a combination of clinical features—including shorter symptom duration, anticoagulant use, and more frequent neurological deficits—that may raise suspicion for CSLEH when conventional MRI findings overlap with those of a lumbar synovial cyst.

Nevertheless, several limitations should be acknowledged. First, the retrospective design may have introduced selection and information bias. Second, the relatively small sample size, particularly in the CSLEH group, reflects the rarity of this condition and limits the statistical power and precision of the between-group comparisons. No a priori sample size or power calculation was performed, and the sample sizes of the individual groups were determined by the number of eligible patients available during the predefined study period. Consequently, the study may have been underpowered to detect smaller but potentially clinically relevant differences, particularly in radiological variables. Therefore, non-significant findings should be interpreted cautiously, and the present results should be regarded as exploratory and hypothesis-generating. Third, this was a single-center study, which may limit the generalizability of the findings. In addition, radiological assessment was not performed by blinded independent reviewers, and interobserver agreement could therefore not be assessed. Owing to the retrospective nature of the study and the lack of standardized imaging protocols across all patients, potentially discriminating features such as contrast-enhancement patterns, detailed lesion morphology, and susceptibility-related blood-product characteristics could not be systematically evaluated. Furthermore, histopathological confirmation was not available in all cases, particularly in patients with chronic spontaneous lumbar epidural hematoma, in whom the diagnosis was established on the basis of characteristic intraoperative findings. Finally, the timing of postoperative neurological assessments was not standardized, and long-term functional and neurological outcomes could not be comprehensively assessed. Therefore, larger multicenter studies with prospectively defined sample size requirements are warranted to validate these findings and further define the distinguishing clinical and radiological features of chronic spontaneous lumbar epidural hematoma and lumbar synovial cyst.

## 5. Conclusions

Chronic spontaneous lumbar epidural hematoma should be considered in the differential diagnosis when a lumbar synovial cyst is suspected, particularly in patients presenting with a relatively short symptom duration, anticoagulant use, rapidly progressive neurological deficits, and inconclusive MRI findings. Although conventional MRI remains indispensable for preoperative evaluation, substantial overlap in MRI characteristics may limit reliable differentiation between these two entities. Careful integration of clinical presentation and MRI findings may aid preoperative differentiation; however, in our cohort, definitive diagnosis was established intraoperatively. Surgical decompression provided favorable clinical outcomes in both groups while simultaneously relieving neural compression and establishing the definitive diagnosis. Further multicenter studies with larger patient cohorts are warranted to validate these findings and better define the clinical and radiological features that distinguish chronic spontaneous lumbar epidural hematoma from lumbar synovial cysts.

## Figures and Tables

**Table 1:** Baseline demographic and clinical characteristics according to final diagnosis.

Variable	CSLEH (*n* = 6)	Synovial Cyst (*n* = 10)	Effect Size (95% CI)	*p* Value
**Age (years), mean ± SD**	66.8 ± 7.3	59.8 ± 11.9	g = 0.63 (−0.35 to 1.62)	0.216
**Female sex, *n* (%)**	3 (50.0)	7 (70.0)	RD = −20.0% (−56.7% to 23.5%)	0.607 ^a^
**Comorbidity present, *n* (%)**	5 (83.3)	6 (60.0)	RD = 23.3% (−22.6% to 55.1%)	0.588 ^a^
**Anticoagulant use, *n* (%)**	5 (83.3)	1 (10.0)	**RD = 73.3% (23.3% to 89.3%)**	**0.008 ^a^**
**Symptom duration (days), mean ± SD**	23.8 ± 12.3	128.5 ± 73.7	**g = −1.66 (−2.80 to −0.52)**	**0.001**
**Motor weakness, *n* (%)**	6 (100.0)	0 (0.0)	**RD = 100.0% (52.1% to 100.0%)**	**<0.001 ^a^**
**Sensory deficit, *n* (%)**	5 (83.3)	0 (0.0)	**RD = 83.3% (34.9% to 97.0%)**	**0.001 ^a^**

Values are presented as mean ± standard deviation or number (%). Continuous variables were compared using independent-samples *t* tests, and categorical variables were compared using Fisher’s exact tests. Effect sizes are presented as Hedges’ g with 95% confidence intervals (CIs) for continuous variables and absolute differences in proportions (RDs) with 95% CIs for categorical variables. CSLEH, chronic spontaneous lumbar epidural hematoma; CI, confidence interval; RD, absolute difference in proportions. ^a^ Fisher’s exact test. Significant *p* values (*p* < 0.05) are shown in bold.

**Table 2:** MRI characteristics and lesion localization according to final diagnosis.

Variable	Chronic Spontaneous Epidural Hematoma (*n* = 6)	Synovial Cyst (*n* = 10)	*p* Value
T1-weighted MRI signal, *n* (%)			0.250 ^a^
Isointense	6 (100.0)	7 (70.0)	
Hypointense	0 (0.0)	3 (30.0)	
T2-weighted MRI signal, *n* (%)			0.125 ^a^
Isointense	1 (16.7)	0 (0.0)	
Hypointense	1 (16.7)	0 (0.0)	
Hyperintense	4 (66.7)	10 (100.0)	
Lesion localization, *n* (%)			0.126 ^a^
T12–L1	1 (16.7)	1 (10.0)	
L1–L2	1 (16.7)	0 (0.0)	
L2–L3	1 (16.7)		
L3–L4	2 (33.3)	1 (10.0)	
L4–L5	1 (16.7)	6 (60.0)	
L5–S1	0 (0.0)	2 (20.0)	

Values are expressed as n (%). Categorical variables were compared using Fisher’s exact test or exact chi-square tests as appropriate. ^a^ Exact *p* values.

**Table 3:** Surgical outcomes and pain improvement according to final diagnosis.

Variable	CSLEH (*n* = 6)	Synovial Cyst (*n* = 10)	Effect Size (95% CI)	*p* Value
Preoperative VAS score	8.5 ± 0.5	6.1 ± 1.0	**Hedges’ g = 2.65 (1.28 to 4.02)**	**<0.001 ^a^**
Postoperative VAS score	0.8 ± 0.8	0.3 ± 0.5	Hedges’ g = 0.76 (−0.24 to 1.75)	0.181 ^a^
VAS improvement (ΔVAS)	7.7 ± 1.2	5.8 ± 0.8	**Hedges’ g = 1.87 (0.69 to 3.05)**	**0.007 ^a^**
Postoperative complication, n (%)	0 (0.0)	0 (0.0)	—	—

Values are presented as mean ± standard deviation. ΔVAS = preoperative VAS − postoperative VAS. Effect sizes are presented as Hedges’ g with 95% confidence intervals (CIs). CSLEH, chronic spontaneous lumbar epidural hematoma; VAS, Visual Analog Scale; CI, confidence interval. ^a^ Mann–Whitney U test. Bold values indicate statistical significance (*p* < 0.05).

**Table 4:** Practical clinical and radiological features that may assist in the preoperative differentiation between chronic spontaneous lumbar epidural hematoma and lumbar synovial cyst.

Feature	Chronic Spontaneous Lumbar Epidural Hematoma (CSLEH)	Lumbar Synovial Cyst
Clinical course	Acute or subacute symptom progression	Chronic, slowly progressive symptoms
Symptom duration	Usually shorter	Usually longer
Anticoagulant use	Frequently present	Less common
Motor deficit	More frequent	Less frequent
Sensory deficit	More frequent	Less frequent
Preoperative pain severity	Usually more severe	Usually less severe
MRI appearance	Variable; considerable overlap with synovial cyst	Variable; considerable overlap with CSLEH
Typical location	Variable; may occur at any lumbar level	Most commonly L4–L5
Diagnostic clue	Consider in patients with anticoagulant use and rapidly progressive neurological deficits despite inconclusive MRI findings	Consider in patients with longstanding degenerative symptoms and facet joint degeneration

## Data Availability

The datasets analyzed during the current study are available from the corresponding author upon reasonable request.
